# Mechanisms of Neurosyphilis-Induced Dementia: Insights into Pathophysiology

**DOI:** 10.3390/neurolint16060120

**Published:** 2024-12-02

**Authors:** Aya Fadel, Hussain Hussain, Robert J. Hernandez, Amanda Marie Clichy Silva, Amir Agustin Estil-las, Mohammad Hamad, Zahraa F. Saadoon, Lamia Naseer, William C. Sultan, Carla Sultan, Taylor Schnepp, Arumugam R. Jayakumar

**Affiliations:** 1 Department of Internal Medicine at Ocean Medical Center, Hackensack Meridian Health, Hackensack, NJ 07601, USA; 2 Department of Internal Medicine, HCA Florida Kendall Hospital, Miami, FL 33175, USA; roberthernandezmd@gmail.com (R.J.H.); zahraa.fadhil0178@gmail.com (Z.F.S.); lamianaseer9@gmail.com (L.N.); 3 School of Medicine, St. George’s University, 3500 Great River, Islip, NY 11739, USA; asilva2@sgu.edu; 4 School of Medicine, Ross University, Miramar, FL 33025, USA; amirestillas@mail.rossmed.edu (A.A.E.-l.); mhamad1@sgu.edu (M.H.); taylor.schnepp916@gmail.com (T.S.); 5 Department of Psychiatry, Broward Health Medical Center, Fort Lauderdale, FL 33316, USA; wsultan@browardhealth.org; 6 Department of Psychiatry, Southern Winds Hospital, Hialeah, FL 33012, USA; cdavila@southernwindshospital.com; 7 Department of Obstetrics, Gynecology and Reproductive Sciences, School of Medicine, University of Miami Miller, Miami, FL 33136, USA; ajayakumar@med.miami.edu

**Keywords:** dementia, TDP-43, syphilis, neurosyphilis, penicillin

## Abstract

Neurosyphilis-induced dementia represents a severe manifestation of tertiary syphilis, characterized by cognitive and neuropsychiatric impairments. This condition arises from the progression of syphilis to the central nervous system, where the spirochete causes damage through invasion, chronic inflammation, and neurodegeneration. The pathophysiology involves chronic inflammatory responses, direct bacterial damage, and proteinopathies. *Treponema pallidum* triggers an inflammatory cascade, resulting in neuronal injury and synaptic dysfunction. Abnormal protein accumulations, including TAR DNA-binding protein 43 (TDP-43) and tau, contribute to neuronal loss and cognitive decline. Seizures, psychiatric symptoms, and motor deficits further complicate the progression of dementia. Diagnosis includes clinical assessment, cerebrospinal fluid analysis, and neuroimaging. Diagnostic tests include CSF-VDRL, FTA-ABS, and neuroimaging techniques such as MRI and PET scans, which help detect structural changes and confirm neurosyphilis. Management of neurosyphilis-induced dementia involves antibiotic therapy and psychotropic medications to address both infectious and symptomatic components. While penicillin remains the cornerstone of treatment, psychotropic agents, including haloperidol, risperidone, quetiapine, and divalproex sodium, can manage psychiatric symptoms. However, careful monitoring is required due to potential side effects and interactions with ongoing treatment. Overall, early diagnosis and comprehensive management are crucial for mitigating the cognitive and neuropsychiatric impairments associated with neurosyphilis-induced dementia.

## 1. Introduction

*Treponema pallidum* (TP), the bacterium responsible for syphilis, is a spirochete bacterium known for its distinctive helical shape and invasive characteristics [1]. As a stealth pathogen, TP effectively evades the host’s immune system, facilitating its persistence and progression in the host [1]. Its unique spiral structure, propelled by flagellar motility, enables the bacterium to penetrate mucous membranes and disseminate through tissues and the bloodstream [1,2]. The pathogen’s minimal surface antigenicity and ability to invade intercellular junctions of endothelial cells allow it to escape immune detection [3,4]. The streamlined genome of TP, adapted to its parasitic lifestyle, lacks many conventional metabolic pathways, relying heavily on the host for survival.

Syphilis is present as a complex, multifaceted disease with distinct stages. Primary syphilis typically involves a painless chancre at the site of infection, followed by secondary syphilis characterized by systemic manifestations such as rash and lymphadenopathy [5]. If untreated, the infection enters a latent phase, eventually progressing to tertiary syphilis, which can involve gummatous lesions, cardiovascular complications, and neurosyphilis [1,2,3]. Congenital syphilis, resulting from maternal transmission, poses significant risks to newborns, including severe abnormalities [5].

Neurosyphilis, a severe complication of untreated syphilis, targets the central nervous system and can manifest in various forms, including meningitis, stroke-like syndromes, and dementia [6]. Neurosyphilis-induced dementia is particularly devastating, affecting cognitive function, behavior, and motor skills [6]. Late-stage symptoms include emotional instability, impaired judgment, memory deficits, and seizures, with some patients developing frank psychiatric disorders such as psychosis and mania [2].

Despite the availability of penicillin, which significantly reduces the prevalence of late-stage syphilis, neurosyphilis continues to pose diagnostic and treatment challenges, particularly in cases of dementia [6]. A 2021 study highlighted the resurgence of syphilis globally, particularly among men who have sex with men (MSM), with the incidence of neurosyphilis remaining a concern [7,8]. Neurosyphilis-induced dementia, though less common today, complicates diagnosis by mimicking other neurodegenerative conditions, leading to potential underrecognition [9].

In this review, we explore the fundamental pathophysiology of neurosyphilis-induced dementia, drawing on current research to clarify its mechanisms and impact. By understanding the relationship between TP infection and neurodegeneration, we aim to inform clinical practices and guide future research in this evolving area.

## 2. Epidemiology

The epidemiology of syphilis and its progression to neurosyphilis leading to dementia reflects a significant public health concern, particularly in specific high-risk populations [1,10,11]. Syphilis incidence has risen globally, with notable increases among MSM and individuals with a history of HIV co-infection [10,11]. Recent data indicate a global prevalence of syphilis in MSM of approximately 7.5% between 2000 and 2020, a stark contrast to the lower prevalence in the general male population [7,10,11]. Additionally, the resurgence of syphilis has been observed in various regions, including Central Sub-Saharan Africa, where the incidence is among the highest worldwide, and the United States, which saw a 10% increase in primary and secondary syphilis cases in 2022 [10,11]. This rise in syphilis cases is accompanied by a concerning increase in congenital syphilis, which poses severe risks to newborns [12]. Neurosyphilis, particularly when untreated, can lead to dementia characterized by cognitive decline and neuropsychiatric symptoms. Epidemiological studies have estimated that up to 5% of individuals with untreated syphilis develop neurosyphilis, with dementia being a frequent manifestation [10,11,13]. The complexity of diagnosing neurosyphilis-induced dementia, often mimicking other neurodegenerative disorders, contributes to underreporting and challenges in accurately assessing its true prevalence [10]. Socioeconomic factors, including limited access to healthcare and preventive measures, further exacerbate the risk and impact of syphilis and its neurological complications [14].

## 3. Pathophysiology

The progression of syphilis through its stages provides insight into the development of neurosyphilis. In primary syphilis, a painless ulcer, or chancre, forms at the site of infection [1]. During secondary syphilis, TP disseminates through the bloodstream, causing systemic symptoms such as rashes and mucocutaneous lesions [15]. If untreated, the infection may enter a latent phase, during which TP can persist in the central nervous system (CNS) [15]. This latent infection can progress to neurosyphilis when the bacterium crosses the blood-brain barrier (BBB), which is more likely to occur during early disseminated or untreated latent syphilis [7,15,16].

**Figure 1:**
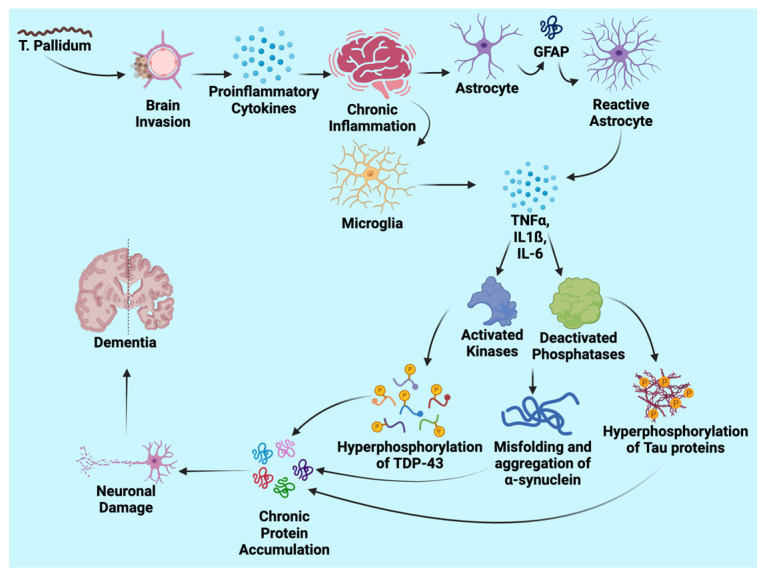
Pathophysiology of *T. pallidum* in the development of dementia.

The pathophysiology of neurosyphilis leading to dementia is a multifaceted process involving chronic inflammation, direct bacterial invasion, and disruptions in protein homeostasis (Figure 1). Neurosyphilis begins when *Treponema pallidum* (TP) enters the body through mucous membranes or breaks in the skin, facilitated by mucopolysaccharidase, which assists in bacterial adherence to host cells [1,2,17,18]. This initial interaction triggers obliterative endarteritis and necrotic changes in terminal arterioles, setting off a cascade of inflammatory responses. These responses are characterized by an infiltration of lymphocytes and plasma cells, resulting in neuronal damage and demyelination [18]. In particular, tabes dorsalis, a severe manifestation of neurosyphilis, involves damage to the dorsal roots and posterior spinal columns, leading to significant neurological deficits [19].

Neurosyphilis manifests in several forms depending on the extent and location of CNS involvement. Asymptomatic neurosyphilis is characterized by the presence of TP in cerebrospinal fluid (CSF) without overt clinical symptoms, with CSF analysis revealing pleocytosis and elevated protein levels [6,20]. Symptomatic meningitis presents with headache, nausea, and photophobia, with CSF showing elevated white cell counts and protein levels [21]. Meningovascular neurosyphilis involves inflammation of the meninges and cerebral blood vessels, leading to conditions such as meningitis and vasculitis [21]. This can result in transient ischemic attacks and ischemic strokes due to compromised blood flow [16,17]. Parenchymal neurosyphilis, in contrast, directly affects brain tissue, leading to neuronal damage and gliosis [22].

In late-stage neurosyphilis, severe CNS damage and neurological deficits become evident [20]. General paresis, or dementia paralytica, is marked by progressive cortical atrophy and neuronal loss [23]. Symptoms include personality changes, emotional instability, delusions, hallucinations, memory loss, and impaired judgment [24]. Tabes dorsalis is characterized by degeneration of the dorsal columns and roots of the spinal cord, resulting in ataxia, sensory deficits, and bladder dysfunction [24,25].

The progression to dementia involves several interrelated mechanisms. Chronic inflammation in the CNS due to TP leads to immune activation, neuronal injury, and synaptic dysfunction [1,4,18]. Vascular inflammation or vasculitis exacerbates ischemic damage by disrupting cerebral blood flow, further contributing to cognitive decline [18]. Direct neural damage from bacterial invasion and its byproducts accelerates neurodegeneration. As TP crosses the BBB, it triggers an inflammatory response that leads to meningitis, vasculitis, and neuronal damage in both cortical and subcortical structures, resulting in cognitive decline, memory impairment, personality changes, and impaired executive functions [18].

Abnormal protein accumulations also play a significant role in neurosyphilis-induced dementia. TAR DNA-binding protein 43 (TDP-43) plays a crucial role in several neurodegenerative diseases, including amyotrophic lateral sclerosis (ALS) and frontotemporal dementia (FTD) [26]. TDP-43 is involved in critical cellular processes such as transcription regulation, RNA splicing, and stress response. However, in these diseases, TDP-43 undergoes pathological changes, including hyperphosphorylation and mislocalization, leading to cytoplasmic aggregates [26]. These abnormal aggregates are thought to contribute significantly to neuronal damage and cell death, resulting in cognitive decline and other neurodegenerative symptoms [26].

In neurodegenerative conditions, TDP-43 aggregates disrupt normal cellular functions [26,27]. The protein becomes hyperphosphorylated and cleaved, forming insoluble aggregates that accumulate in the cytoplasm [27]. These aggregates interfere with RNA processing, sequester other RNA-binding proteins, and induce cellular stress responses [26,27]. The resulting toxicity from TDP-43 aggregates contributes to neuronal dysfunction and death, exacerbating neurodegenerative processes and cognitive decline [26,27].

Tau protein abnormalities, including hyperphosphorylation and the formation of neurofibrillary tangles, are implicated in the pathology of neurodegenerative diseases and contribute to neuronal damage [28,29]. Though less commonly associated, alpha-synuclein aggregates may also play a role in affecting synaptic function [28].

The neuroinflammatory response in neurosyphilis involves the activation of microglia, which release pro-inflammatory cytokines such as tumor necrosis factor-alpha (TNF-α), interleukin-1 beta (IL-1β), and interleukin-6 (IL-6). Also, chronic microglial activation can lead to reactive oxygen species (ROS) production [18,26,30]. This inflammatory milieu exacerbates neuronal damage and protein aggregation, creating a neurotoxic environment that accelerates cognitive decline [18,26,30]. Furthermore, vascular damage and chronic inflammation may influence amyloid precursor protein (APP) processing, potentially exacerbating amyloid deposition, though this connection is less well-documented in neurosyphilis [18,26,30].

Astrocytes are another critical type of glial cell involved in neurosyphilis-induced dementia [31]. They support neuronal function by maintaining the extracellular environment, regulating neurotransmitter levels, and providing metabolic support [18]. During neurosyphilis, astrocytes become reactive in response to TP-induced inflammation [18,30]. This reactive astrogliosis involves the upregulation of glial fibrillary acidic protein (GFAP) and changes in astrocyte morphology [18]. Reactive astrocytes contribute to neuroinflammation by releasing inflammatory mediators and cytokines, which can further damage neurons [18,22]. They also play a role in accumulating neurotoxic protein aggregates, such as beta-amyloid and tau, by failing to clear these proteins from the extracellular space effectively [22]. The disruption of astrocyte function impairs synaptic plasticity and cognitive function, contributing to the progression of dementia [18].

When co-infection with HIV occurs, the pathophysiology of neurosyphilis-induced dementia becomes more complex [32]. HIV affects the CNS through direct viral invasion and an inflammatory response. The virus crosses the BBB, infecting brain macrophages and microglia, leading to chronic neuroinflammation [32]. Key inflammatory mediators, including TNF-α and IL-1β, contribute to neuronal injury and neurodegeneration [32]. HIV proteins, such as gp120 and Tat, exacerbate neuronal damage by increasing oxidative stress and excitotoxicity and by disrupting synaptic plasticity [32,33].

The presence of both HIV and TP leads to a compounded neurodegenerative process. The combined effects of viral and bacterial inflammation result in severe neuroinflammation and oxidative stress, exacerbating neuronal damage and accelerating cognitive impairments [32]. The presence of TDP-43 and tau protein abnormalities in both infections contributes to a more severe neurodegenerative phenotype [32,33]. HIV-induced vasculitis and syphilitic inflammation of cerebral blood vessels lead to compromised blood flow and ischemic injury, further disrupting neurovascular function and contributing to cognitive decline [33].

The cholinergic system, essential for learning, memory, and attention, is often disrupted in neurodegenerative conditions. In neurosyphilis, TP infection may contribute to cholinergic deficits by causing inflammatory and neurodegenerative changes in brain regions rich in cholinergic neurons, potentially leading to symptoms like memory loss, impaired judgment, and personality changes [34]. While neurosyphilis does not directly cause these proteinopathies, chronic infection-induced neuroinflammation might create an environment conducive to amyloid deposition and tau hyperphosphorylation, contributing to cognitive decline. Exploring these parallels can highlight potential overlapping pathways in neurosyphilis-related and Alzheimer’s dementia. Further, NMDA receptor dysregulation is linked to excitotoxicity and neuronal damage in various dementias. Neurosyphilis may similarly disrupt NMDA signaling through inflammatory pathways, exacerbating cognitive dysfunction [35]. A thorough examination of these mechanisms could enhance understanding of neurosyphilis-induced dementia, potentially guiding targeted treatments that address not only the infection but also the neurological sequelae.

The mechanism by which TP induces neuroinflammation is not fully understood, as TP lacks the endotoxins typically associated with bacterial inflammation, such as LPS found in gram-negative bacteria. However, TP may release specific excretory products, including lipoproteins and outer membrane proteins, that could stimulate inflammatory pathways in the brain [36]. Identifying these excretory factors is crucial, as they may be directly responsible for the neuronal damage observed in neurosyphilis.

## 4. Clinical Manifestation

Neurosyphilis-induced dementia is a multifaceted condition resulting from the advanced stages of syphilis, characterized by diverse clinical features reflecting significant central nervous system (CNS) involvement. One of the hallmark manifestations is profound cognitive decline [1,37]. Patients typically experience severe memory deficits, particularly in short-term and recent memory [37]. Challenges in attention and concentration accompany this mental impairment. As the disease progresses, executive functions, including planning, problem-solving, and organizational skills, become markedly impaired [38].

Behavioral and personality changes are also prominent in neurosyphilis-induced dementia [32]. Individuals may undergo significant shifts in personality, displaying emotional instability and erratic behavior [25,38]. This can manifest as irritability, mood swings, and socially inappropriate actions. Emotional lability, marked by sudden shifts between euphoria and depression, is frequently observed [39]. In addition, psychiatric symptoms further complicate the clinical picture. Patients may experience delusions, hallucinations, and paranoia [32]. Psychotic symptoms, including detachment from reality and severe mood disturbances such as mania or profound depression, are common [32]. These psychiatric manifestations can mimic other psychiatric disorders, adding complexity to diagnosis and treatment [28].

One prominent clinical feature of TP infection is the development of seizure disorders [40]. The progressive cortical damage and inflammation associated with neurosyphilis can disrupt regular brain activity, increasing the risk of both focal and generalized seizures [40]. Seizures can exacerbate cognitive decline and complicate the clinical management of the disease [40].

Motor and neurological deficits are critical aspects of neurosyphilis-induced dementia. Patients may present with tremors, ataxia (lack of coordination), and dysarthria (difficulty articulating speech) [41]. Gait disturbances, muscle weakness, and coordination problems are also prevalent, reflecting the direct impact of TP on neural structures and the resultant neuronal damage [41]. Cognitive-motor integration issues are observed, with patients struggling with multitasking and coordinating complex activities, significantly impairing daily functioning [41].

Parenchymatous syphilis, a severe manifestation of tertiary syphilis, primarily affects the CNS and cardiovascular system, presenting a range of clinical features. This late stage of syphilis can be categorized into two distinct forms: paretic neurosyphilis and tabetic neurosyphilis, each with unique symptoms and pathological characteristics [42].

Paretic neurosyphilis, also known as general paresis, is characterized by a progressive decline in cognitive and emotional functioning [42,43]. Early symptoms include irritability, forgetfulness, personality changes, headaches, and disturbances in sleep patterns [43]. As the disease advances, patients may exhibit more severe symptoms such as emotional lability, impaired memory and judgment, disorientation, confusion, delusions, and, in some cases, seizures [40,41,42,43]. These manifestations arise from the infiltration of TP into the gray matter of the brain and its effects on endothelial and microglial cells, which contribute to the observed cognitive and emotional disturbances [42]. Pupillary abnormalities, while occasionally present, are less common in paretic neurosyphilis compared to tabetic forms [43].

The degeneration of the posterior roots and columns of the spinal cord characterizes tabetic neurosyphilis [44]. This form presents various sensory and motor symptoms, including an ataxic gait, lightning pains, paresthesias, bladder dysfunction, and vision impairment [43,44]. Notable clinical features include pupillary abnormalities, with Argyll Robertson pupils being the most characteristic finding [45]. Patients may also exhibit diminished reflexes, impaired vibratory sense and proprioception, ocular palsies, and Charcot’s joints—pathological conditions resulting from joint degeneration due to loss of proprioceptive feedback [44,45].

In addition to CNS manifestations, parenchymatous syphilis can lead to significant cardiovascular complications, including aortitis and the formation of aortic aneurysms [46]. These complications pose serious risks, such as aortic regurgitation or dissection, further complicating the disease and underscoring the critical need for early diagnosis and intervention [46,47].

As neurosyphilis-induced dementia progresses, patients may experience significant functional impairments [48]. These include difficulties with daily living activities such as personal care and household tasks. The combined effect of cognitive decline, motor deficits, and behavioral changes can lead to a profound loss of independence, necessitating increased caregiver support and potential institutionalization [48].

## 5. Diagnosis

The diagnosis of neurosyphilis-induced dementia involves a comprehensive and multidisciplinary approach, integrating clinical evaluation, laboratory testing, neuroimaging, and cognitive assessments [44]. Given the complex nature of neurosyphilis and its potential overlap with other neurodegenerative conditions, a thorough diagnostic process is essential.

Clinical evaluation is a fundamental component of diagnosing neurosyphilis-induced dementia. It begins with a detailed patient history, including information about previous syphilis diagnoses, treatments, sexual history, and any neurological symptoms experienced [44,49]. A neurological examination follows this to assess for signs of cranial nerve dysfunction, motor and sensory abnormalities, gait disturbances, and cognitive deficits. Typical findings in neurosyphilis include changes in mental status, memory impairment, personality changes, and motor symptoms such as tremors and ataxia [44,49].

Laboratory testing plays a crucial role in the diagnostic process. Cerebrospinal fluid (CSF) analysis is central to diagnosing neurosyphilis. Key tests include the following:

- CSF Cell Count: Elevated white blood cell count, or pleocytosis, can indicate inflammation [50,51,52].
- Protein Levels: Increased protein levels in CSF may suggest an inflammatory process [50,51,52].
- CSF-VDRL Test: A positive result is highly suggestive of neurosyphilis, though false negatives can occur [50,51,52].
- FTA-ABS and TP-PA Tests detect specific antibodies to *Treponema pallidum* in CSF. The FTA-ABS test is highly sensitive for detecting neurosyphilis, while the TP-PA test helps confirm the presence of these antibodies [52].

Serological tests are also crucial for diagnosing syphilis:

- Non-treponemal Tests: These include RPR (Rapid Plasma Reagin) and VDRL (Venereal Disease Research Laboratory) tests, which detect nonspecific antibodies and can indicate active infection [50,51,52].
- Treponemal Tests, such as FTA-ABS (Fluorescent Treponemal Antibody Absorption), which detect specific antibodies against *Treponema pallidum* [50,51,52].

**Figure 2:**
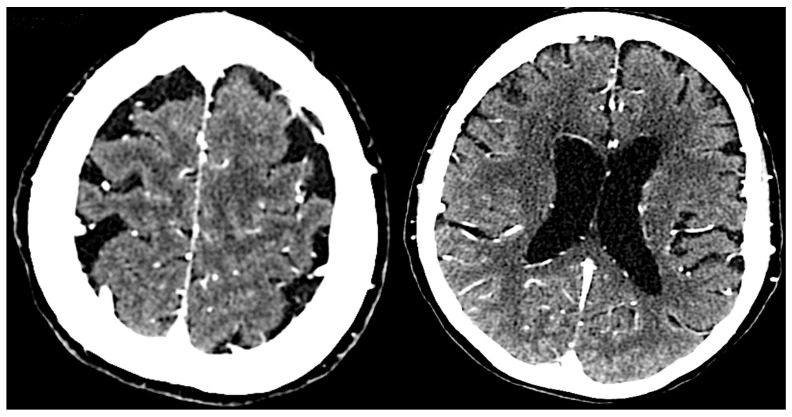
The brain CT scan shows cerebral atrophy with slight ventricular enlargement.

Neuroimaging supports the diagnosis by revealing structural changes in the brain associated with neurosyphilis (Figure 2). Magnetic Resonance Imaging (MRI) can show cortical atrophy, white matter lesions, and vascular abnormalities. Specific findings, such as hydrocephalus or lesions in the basal ganglia, can suggest neurosyphilis-related pathology [50,51,52]. Computed Tomography (CT) scans also identify significant structural abnormalities and rule out other causes of neurological symptoms, such as tumors or hemorrhages [52].

Cognitive and neuropsychological testing is crucial for assessing the impact of neurosyphilis on cognitive function [48]. Cognitive assessments evaluate various domains, including memory, attention, executive function, and language skills [48]. In neurosyphilis-induced dementia, patients often exhibit deficits in memory, executive functions, and attention [48]. Functional assessments are used to evaluate how cognitive deficits affect daily functioning, which helps in determining the severity of dementia and guiding treatment strategies [48]. Standardized cognitive assessments, such as the Mini-Mental State Examination (MMSE) and the Montreal Cognitive Assessment (MoCA), evaluate a range of cognitive functions, including memory, attention, and executive abilities [48]. These tests help identify impairments across various cognitive domains indicative of dementia.

## 6. Differential Diagnosis

It is essential to distinguish neurosyphilis-induced dementia from other forms of dementia and neurodegenerative disorders. Alzheimer’s disease is characterized by amyloid plaques and neurofibrillary tangles, which are not typically associated with neurosyphilis [51]. Frontotemporal dementia, marked by early onset personality changes and behavioral symptoms, may overlap with neurosyphilis symptoms but has different underlying pathologies [51,52]. HIV-associated dementia, which can present similarly to neurosyphilis-induced dementia, is differentiated by the presence of HIV infection and associated neuroinflammatory markers [51].

Differentiating neurosyphilis-related dementia from other forms of dementia relies on specific clinical, laboratory, and imaging findings that suggest TP infection in the central nervous system. Unlike Alzheimer’s disease, which is primarily characterized by memory impairment and a gradual cognitive decline, neurosyphilis-related dementia often presents with prominent behavioral and personality changes early on, such as mood swings, irritability, and paranoia [37,41]. Patients may also exhibit characteristic signs like Argyll Robertson pupils (pupils that accommodate but do not react to light) and focal neurological deficits, which are uncommon in other dementias [37]. Laboratory tests, including CSF analysis, play a crucial role. In neurosyphilis, the CSF typically shows pleocytosis (increased white blood cells), elevated protein, and positive syphilis-specific tests, such as the CSF-VDRL (Venereal Disease Research Laboratory) test [37,53]. While less definitive, imaging studies may show cortical atrophy or white matter changes that are not specific but can support the diagnosis when combined with clinical and CSF findings [53].

## 7. Treatment

The management of persistent dementia resulting from neurosyphilis involves a comprehensive approach that includes addressing the underlying infection and managing ongoing cognitive and psychiatric symptoms. Effective treatment typically combines antimicrobial therapy with symptomatic management, utilizing various medications tailored to individual patient needs.

Antibiotic Therapy: The primary treatment for neurosyphilis is high-dose intravenous penicillin G, which is crucial for eliminating *Treponema pallidum* and preventing disease progression [53]. The standard regimen involves 18–24 million units per day, administered intravenously in divided doses over 10–14 days [53]. This intensive therapy penetrates the central nervous system and addresses latent infections that may contribute to persistent symptoms.

Symptomatic Management: For patients with persistent symptoms after adequate antibiotic treatment, addressing residual cognitive and psychiatric symptoms is essential [54]. This often involves the use of psychotropic medications.

Antipsychotic Medications: Antipsychotic medications are employed to manage severe psychosis and agitation. Haloperidol, a typical antipsychotic, can be used at doses ranging from 1–10 mg per day based on symptom severity and patient tolerance [55,56,57,58]. Atypical antipsychotics, such as risperidone and quetiapine, are commonly preferred due to their improved side effect profiles [55,56,57,58]. Risperidone is usually dosed at 1–4 mg daily, while quetiapine is administered at 50–300 mg per day, adjusted according to clinical response [56,57].

Mood Stabilizers: Divalproex sodium, used for mood stabilization and treatment of agitation, is typically initiated at 250–500 mg twice daily [56,58]. The target therapeutic plasma level for divalproex sodium is 50–100 µg/mL, with doses adjusted accordingly [58]. However, evidence supporting its efficacy in dementia-related agitation is mixed, and alternative treatments may be considered if the response is insufficient [57].

Cognitive Enhancers: In cases where standard treatments are ineffective, additional medications used to manage dementia may be considered. Cholinesterase inhibitors, such as donepezil, rivastigmine, and galantamine, are commonly used to manage cognitive symptoms in various types of dementia [34]. Donepezil is typically dosed at 5–10 mg daily, rivastigmine at 1.5–6 mg twice daily, and galantamine at 8–24 mg daily, adjusted based on tolerability and clinical response [34].

NMDA Receptor Antagonists: Memantine, an NMDA receptor antagonist, may be used to address persistent cognitive symptoms [59]. It is generally administered at an initial dose of 5 mg daily, increasing to a maximum of 20 mg per day as tolerated. Memantine can help manage moderate to severe dementia symptoms by regulating glutamatergic activity [59].

Cognitive and Behavioral Interventions: Cognitive rehabilitation and behavioral therapies may also be beneficial in managing persistent symptoms [60]. These therapies aim to improve cognitive function and manage behavioral disturbances, though evidence specific to neurosyphilis-induced dementia remains limited [60].

Monitoring and Adjustments: Ongoing tracking of medication efficacy and side effects is crucial, especially given the potential for adverse reactions and interactions. The lowest effective doses should be utilized, and adjustments should be made based on clinical response. Periodic attempts to reduce or withdraw medications may be appropriate to minimize long-term side effects.

Current Approaches to Anti-TDP-43 Therapy: Several therapeutic strategies are being explored to target TDP-43 pathology [61]. One approach involves using small-molecule inhibitors such as PRM-151, which aims to reduce TDP-43 aggregation and improve neuronal survival [61]. Preclinical studies have shown promise, and clinical trials are underway to evaluate its efficacy and safety in humans [61].

Gene silencing technologies, such as antisense oligonucleotides (ASOs), represent another strategy [62]. ASOs are designed to specifically bind to TDP-43 mRNA, leading to its degradation and reducing TDP-43 protein levels. Initial studies have demonstrated that ASOs can effectively lower TDP-43 levels in animal models, and they are currently being tested for safety and efficacy in human clinical trials [62]. RNA interference (RNAi) techniques, which use small interfering RNAs (siRNAs) to target TDP-43 mRNA, are also under investigation for their potential to reduce TDP-43 expression and alleviate neurodegenerative symptoms [62].

Monoclonal antibodies targeting TDP-43 are another promising approach. These antibodies are designed to recognize and neutralize TDP-43 aggregates, facilitating their clearance from the brain [61]. While still in preclinical and early clinical stages, these therapies offer a potential method for targeting and removing TDP-43 aggregates associated with neurodegenerative diseases [61].

Clinical Challenges and Considerations: Developing effective anti-TDP-43 therapies presents several challenges. One primary concern is the potential for off-target effects and the risk of disrupting normal TDP-43 functions. Additionally, delivering these therapies across the blood-brain barrier remains a significant hurdle. Ongoing research aims to address these challenges, optimize therapeutic strategies, and improve the targeting of TDP-43 pathology in neurodegenerative diseases.

Antibiotics, particularly penicillin, are crucial in the treatment of neurosyphilis to prevent the development of dementia and other severe neurological complications. Intravenous penicillin is the gold standard therapy, as it effectively eradicates the bacteria from the brain and spinal cord, halting the progression of neurological damage. Early intervention with penicillin not only resolves the infection but can also reverse or prevent cognitive decline, offering the best chance to avoid permanent dementia associated with advanced neurosyphilis.

This study opens avenues for exploring more detailed mechanisms underlying neurosyphilis-induced dementia, particularly cholinergic dysfunction, amyloid-beta aggregation, tau pathology, and NMDA receptor signaling. Future studies could investigate the extent to which neurosyphilis influences these pathways at a molecular level and whether interventions targeting these mechanisms can mitigate cognitive decline. Moreover, longitudinal studies assessing cognitive outcomes after early and late treatment of neurosyphilis would be valuable in understanding the timing and efficacy of therapeutic interventions. Research into neuroimaging markers and cerebrospinal fluid biomarkers could also enhance early detection and differentiation of neurosyphilis dementia from other neurodegenerative diseases. The primary limitations of this study include a lack of direct biochemical analysis of cholinergic and NMDA receptor activity in neurosyphilis patients, limiting insight into specific receptor-level interactions. Additionally, the study may be constrained by a small sample size or lack of control groups with other forms of dementia, which could limit the generalizability of the findings. Variability in neurosyphilis presentations, which can range from asymptomatic to severe dementia, also presents a challenge in establishing uniform diagnostic and therapeutic guidelines. Future studies with more extensive diverse populations and more advanced molecular and imaging tools are needed to address these limitations and validate the findings further.

## 8. Preventing Neurosyphilis-Induced Dementia

Early diagnosis and intervention are critical in preventing the progression of neurosyphilis to dementia. Neurosyphilis-induced dementia arises primarily through inflammatory processes within the central nervous system, often leading to irreversible damage marked by tau and amyloid-beta accumulation. Once neuroinflammatory processes begin, they can set off a cascade leading to synaptic and neuronal dysfunction, making reversing or removing neurotoxic proteins and tangles extremely challenging.

Screening high-risk individuals, especially those with early signs of syphilis, could significantly reduce the risk of neurosyphilitic dementia. Identifying neurosyphilis in its early stages, such as during primary or secondary syphilis, enables timely intervention, often with penicillin, to eradicate TP before it invades the CNS. Preventing the transition to late latent syphilis is essential, as this is when the bacterium may become dormant within the CNS, evading immune detection and treatment. Comprehensive treatment at these early stages minimizes the risk of neuroinflammation and associated dementia.

Future research should explore improved methods for early detection, including serological and CSF markers, that can identify CNS invasion before cognitive symptoms appear. Additionally, enhancing public health initiatives for routine syphilis screening, particularly in populations with high risk of transmission, can aid in timely diagnosis and treatment.

## 9. Future Scope and Limitations

This study opens avenues for exploring more detailed mechanisms underlying neurosyphilis-induced dementia, particularly cholinergic dysfunction, amyloid-beta aggregation, tau pathology, and NMDA receptor signaling. Future studies could investigate the extent to which neurosyphilis influences these pathways at a molecular level and whether interventions targeting these mechanisms can mitigate cognitive decline. Moreover, longitudinal studies assessing cognitive outcomes after early and late treatment of neurosyphilis would be valuable in understanding the timing and efficacy of therapeutic interventions. Research into neuroimaging markers and cerebrospinal fluid biomarkers could also enhance early detection and differentiation of neurosyphilis dementia from other neurodegenerative diseases. The primary limitations of this study include a lack of direct biochemical analysis of cholinergic and NMDA receptor activity in neurosyphilis patients, limiting insight into specific receptor-level interactions. Variability in neurosyphilis presentations, which can range from asymptomatic to severe dementia, also presents a challenge in establishing uniform diagnostic and therapeutic guidelines. Future studies with more prominent diverse populations and advanced molecular and imaging tools are needed to address these limitations and further validate the findings.

## 10. Materials and Methods

Search Strategy: To explore the connection between neurosyphilis and dementia, we conducted a comprehensive literature review using PubMed. Our search covered publications from 1990 to 2024, focusing on terms such as “neurosyphilis”, “neurosyphilis-induced dementia,” “syphilis and neuroinflammation”, and “cognitive impairment in neurosyphilis”.

Selection of Studies: Our search identified over 316 relevant manuscripts discussing neurosyphilis and cognitive impairment. Studies were included if they addressed cognitive decline, neuroinflammation, or clinical features of neurosyphilis. We excluded studies without direct clinical relevance, review articles with limited data, and non-human model studies.

Neuroinflammation Mechanisms: We conducted an additional review of recent manuscripts on neuroinflammation caused by TP, focusing on studies that examined inflammatory responses in the brain, such as microglial activation and cytokine release. This subset included more than 10 manuscripts discussing how TP infection might contribute to chronic brain inflammation and the protein buildup associated with dementia.

Outcome Measures: The main focus was to compile a detailed profile of cognitive impairment in neurosyphilis, with an additional emphasis on identifying inflammatory processes that may contribute to neurodegenerative changes. This approach aimed to clarify both established knowledge and research gaps related to neurosyphilitic dementia.

## 11. Conclusions

The bacterium (TP) responsible for syphilis has the unique ability to persist within the CNS, even after initial infection phases have resolved. This persistence can lead to chronic, low-grade inflammation that contributes to gradual neurodegeneration. TP evades the immune response through several mechanisms, including crossing the BBB and unique cellular properties, which help it remain undetected by immune cells. This capability creates a subtle, ongoing inflammatory state within the CNS that may initially be asymptomatic but progressively fosters neuroinflammation and neuronal damage. As TP continues to reside within the CNS, its persistent inflammation may lead to pathological protein accumulation, including tau and beta-amyloid. These proteins disrupt neuronal function, impair synaptic connections, and accelerate neurodegeneration. The chronic inflammatory response activates microglial cells and releases pro-inflammatory cytokines, exacerbating neuronal damage and contributing to neurotoxic protein aggregates. Over time, these changes can significantly impair cognitive function, potentially leading to neurosyphilis-induced dementia. While this understanding of TP’s role in neuroinflammation and neurodegeneration is developing, further research is needed to clarify how TP’s excretory products or cellular interactions contribute to neurodegenerative processes. Specific studies could focus on identifying the molecular mechanisms that link TP’s persistence in the CNS with the formation of neurotoxic protein aggregates and chronic inflammation to better define therapeutic targets for neurosyphilis-associated neurodegeneration.

## Data Availability

Not applicable.
